# Microenvironment Matters: Destabilization of Iridium Anode Catalyst by CO Reduction Products

**DOI:** 10.1021/jacs.5c22283

**Published:** 2026-02-19

**Authors:** Attila Kormányos, Mohd Monis Ayyub, Bence Kutus, Monaza Rashid, Tatiana Priamushko, Gergely F. Samu, Serhiy Cherevko, Balázs Endrődi, Csaba Janáky

**Affiliations:** ∇ Department of Physical Chemistry and Materials Science, 37442 University of Szeged, Rerrich square 1, Szeged H-6720, Hungary; ‡ Department of Molecular and Analytical Chemistry, 37442 University of Szeged, Dóm square 7-8, Szeged H-6720, Hungary; § Forschungszentrum Jülich GmbH, 557193 Helmholtz-Institute Erlangen-Nürnberg for Renewable Energy (IET-2), Cauerstraße 1, 91058 Erlangen, Germany; ∥ MTA-SZTE Lendület “Momentum” Applied Electrochemistry Research Group, 37442 University of Szeged, Rerrich square. 1, H-6720 Szeged, Hungary; ⊥ Interdisciplinary Excellence Center, 37442 University of Szeged, Rerrich square 1, Szeged H-6720, Hungary

## Abstract

Iridium is one of the most frequently employed anode electrocatalysts in CO_2_ and CO electrolysis, thanks to its reasonable overpotential for the oxygen evolution reaction (OER) and high stability under operating conditions. The latter has been challenged recently by a handful of studies where destabilization of iridium was observed, which was explained solely by thermodynamics (iridium is unstable at strong alkaline pH and OER potentials). In this study, we demonstrate that liquid CO and CO_2_ electrolysis products (such as ethanol and acetate) crossing over to the anode side under long-term operation have a severe effect on the stability of iridium. Its dissolution was studied by both ex-situ inductively coupled plasma mass spectrometry (ICP-MS) and in situ (online ICP-MS) techniques. Based on our electrolysis experiments carried out in a broad pH range (pH = 4–14), ethanol, and its partially oxidized counterpart, acetaldehyde, decreases the stability of the anode catalyst. Ethanol/acetaldehyde oxidation competes with the OER and starts in conjunction with the surface oxidation of the Ir catalyst particles. The oxygenated species are consumed by the alcohol/aldehyde oxidation process, preventing the formation of a passivating surface oxide layer, resulting in an increased iridium dissolution rate.

## Graphical abstract

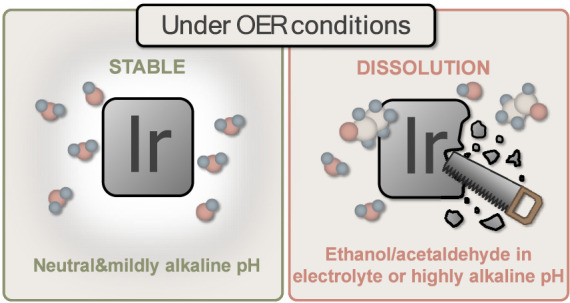

## Introduction

Carbon monoxide and formate can already be produced selectively at industrially relevant reaction rates via low-temperature CO_2_ electrolysis (CO2RR). However, the formation of more complex, C_2+_ products is rather challenging. Further reducing the CO formed in CO2RR in a second electrochemical step could lead to a higher C_2+_ production rate and selectivity (Faradaic efficiency (FE)) due to favorable kinetics and surface coverages of *H and *CO intermediates.^1^ Unlike CO2RR, the electrochemical reduction of CO (CORR) does not suffer from reactant loss due to carbonate formation and crossover,^2^ which ultimately forms a bicarbonate buffer in the recirculated anolyte, posing a challenge for the anode catalysts.^3–5^ The C_2+_ product palette includes gas phase hydrocarbons (e.g., ethylene) and different liquid products, most importantly, acetate and ethanol. These species might pass through the typically used anion exchange membranes (AEMs) in large quantities.^6^ While membrane-separated continuous-flow electrolyzers can be operated in single-pass (flow-through) mode, this, due to the dilute product streams, bears little practical relevance. Recirculating the electrolyte helps the accumulation of liquid phase products.^7,8^ These concentrated product streams can be processed (and separated) further. The presence of CORR products in the anolyte, however, opens the possibility of their oxidation at the anode.

The main structural features of AEMs are the hydrophilic cationic channels that aid the transport of anions through the membrane.^9^ In CORR, negatively charged OH^–^ and acetate ions are generated at the cathode, and the transport of these through the AEM maintains the ionic conductivity between the electrodes. Such AEMs cannot fully prevent the crossover of neutral species (alcohols), which is dictated by the electroosmotic drag and diffusion.^10^ The rate and degree of crossover depend on the cell configuration (i.e., presence of a catholyte) and the operational parameters, but sooner or later, a considerable amount of CORR products gather at the anode side. For example, in our earlier study, we found that almost 70% of the formed ethanol and n-propanol and 100% (!) of the formed acetate crossed over to the anode side in a zero-gap electrolyzer,^6^ similarly to other reports.^9,11^

Organic species (e.g., ethanol, formate, formaldehyde, etc.) can be readily oxidized at the anode at the potentials applied during the oxygen evolution reaction (OER).^7,11–14^ In certain cases, the partial oxidation of these cathode products can be economically beneficial. For example, ethanol that crossed over to the anode side was fully converted into acetate in a CO electrolyzer cell.^7^ Another potential benefit can be the lowered cell voltage, rooted in the notably less positive oxidation potential of these organic molecules compared to OER. These advantages, however, are only temporary; mostly because of the changes occurring in the anolyte composition. A specific example is the decrease of the anolyte pH, which occurs due to OH^–^ consumption during ethanol oxidation to acetate.^15^ The decrease in pH initiates the dissolution of Ni, another commonly used anode for CORR.^16^

The acetate buffer that develops in the anolyte during CO reduction poses a set of new challenges to the anode catalyst. Based on thermodynamic considerations, Ir is a suitable anode catalyst, being fairly stable and active at near-neutral pH.^17^ Interestingly, the instability of Ir was found in former studies employing CO_(2)_ electrolyzer cells.^12,15^ Operando wide-angle X-ray scattering (WAXS) measurements under CORR conditions uncovered considerable Ir dissolution. The dissolved Ir was found in the membrane and also deposited on the cathode catalyst, leading to a decreased CORR selectivity. The accumulation can be circumvented by maintaining a constant anolyte pH, but this is a very energy-, and cost-intensive process. An ultimate solution would be to identify catalysts that are stable under the conditions developing during long-term electrolysis protocols.^3,18^

In the past few years, special attention was dedicated to understanding the stability of Ir under OER conditions, most importantly under conditions developing in PEM water electrolyzers (acidic pH).^19–21^ These studies were performed both in model systems and in cells that are closer to practical application scenarios.^22,23^ Recently, Ir stability was also scrutinized in a broader pH range, identifying Ir overoxidation under alkaline conditions (IrO_4_^2–^ formation) as the main mechanism for Ir dissolution.^17^ While liquid product crossover during CO_2_ or CO electrolysis is common knowledge, the effect of such organic molecules on the stability of the anode catalyst is much less investigated, with only a handful of studies presented in literature so far.^24–29^

In this study, we demonstrate that liquid CORR (and CO2RR) products have a decisive influence on the stability of Ir. Ir dissolution was studied both ex situ (inductively coupled plasma mass spectrometry (ICP-MS)) and in situ (online ICP-MS), employing three different types of continuous flow electrolyzer cells. Control experiments revealed that Ir dissolution increased in the presence of ethanol, scaling with its concentration, regardless of the pH of the anolyte. Further analysis of the anolyte revealed small Ir/IrO_*x*_ nanoparticles leading to a change in the anolyte color. Finally, we also show that a similar phenomenon occurs in CO_2_ electrolyzers too, in agreement with a recent report.^28^

## Results and Discussion

### The Phenomena

As CO electrolysis is inspired directly by CO_2_ electrolysis, most often identical cells, cell components, and catalysts are applied. When performing CO electrolysis in an AEM-separated zero-gap electrolyzer applying copper and iridium as cathode and anode catalysts (see all experimental details in the Supporting Information), however, a monotonous decrease in the CORR product selectivity was observed. In parallel, HER rapidly becomes the dominant cathode process (Figure 1A, B). The voltage change is more complex – first it increases rapidly, plateauing around 2.85 V, followed by its gradual decrease (in about 3 h) to 2.42 V. One reason can be that Ir starts dissolving at the beginning of the electrolysis process (due to the highly alkaline pH) and the OER conditions (see also Figure 1C). The dissolved species get in the membrane, decreasing its conductivity. When these species reach the cathode, Ir deposition occurs (proved by EDX data in Figure S1), shifting the cathode selectivity from CORR to HER. Subsequently, cell operation stabilizes, resulting in a water splitting cell.^30,31^ While these agree with a previous study,^15^ the reason behind the eventually stabilized cell operation, and the rapid Ir dissolution is yet unclear. Notably, a stable operation (at a reasonable cell voltage of ca. 2.3 V) was witnessed in an identically assembled cell, when the cathode was purged with argon instead of CO (brown trace in Figure 1A).

**Figure 1:**
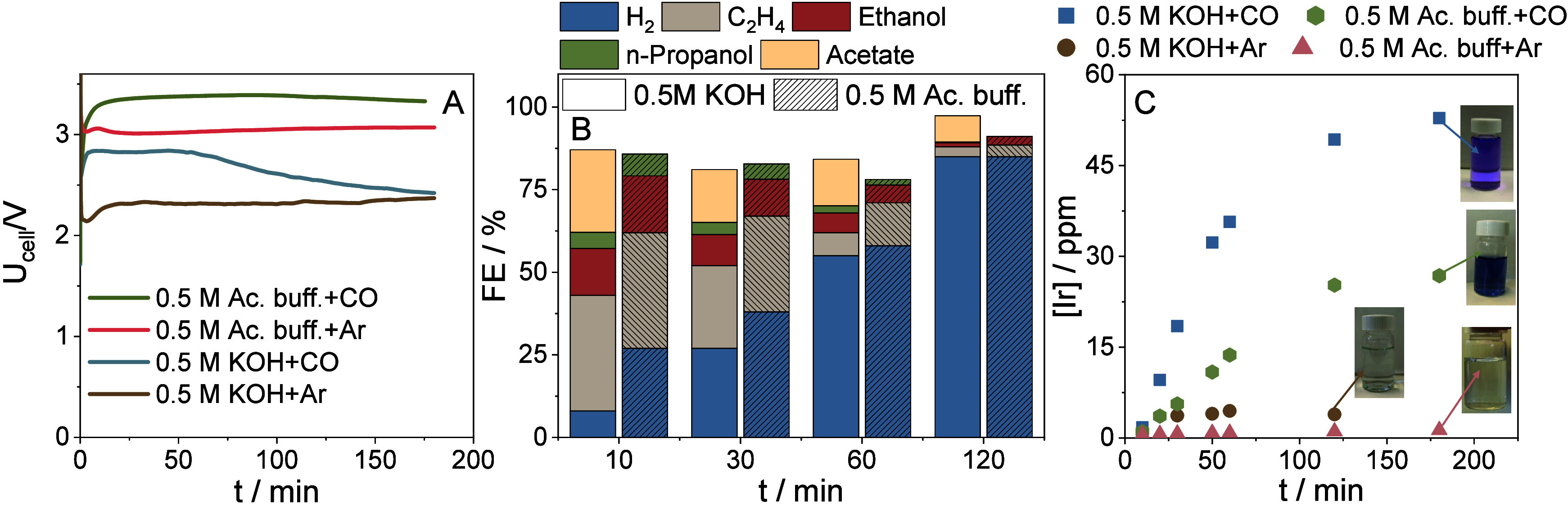
(A) Galvanostatic electrolysis results at j = 200 mA cm^–2^ recorded in 0.5 M KOH or 0.5 M pH = 4.7 acetate buffer anolyte (V = 40 cm^3^, 60 cm^3^ min^–1^ flow rate). The electrolysis experiments were performed in the zero-gap cell configuration. Nonhumidified CO or Ar was fed to the cathode side with a flow rate of 100 cm^3^ min^–1^. (B) Product distribution for the 0.5 M KOH+CO and 0.5 M pH = 4.7 acetate buffer+CO case monitored during the electrolysis measurement (products were collected from both the cathode and anode side of the cell). (C) The amount of iridium dissolved in the liquid-phase aliquots taken during the experiment presented in (A) and analyzed by ICP-MS. Images in the inset show the color of the anolyte at the end of each electrolysis protocol.

Besides CORR selectivity loss, the color of the anolyte gradually changed from transparent to light purple. To see whether the color change is due to Ir dissolution from the anode catalyst layer, samples extracted during the measurement were analyzed by ex-situ ICP-MS (Figure 1C). The presence of Ir was already confirmed after 10 min, and its amount increased with the electrolysis time, following a saturation-type curve. This can be attributed to the increased Ir dissolution in the presence of CORR products. When the selectivity of the cathode process shifts to HER from CORR, CORR product crossover to the anode side also terminates. This decreases the amount of dissolving Ir considerably, hence the plateauing of the [Ir] vs t curve. Interestingly, considerably less Ir was detected in the KOH anolyte when Ar was purged at the cathode, indicating a change in the local chemical environment (no CORR product flux reaches the anode catalyst). Here, the stability of Ir is dictated by the local pH (that possibly deviates from the highly alkaline bulk value),^32,33^ hence the lower Ir concentration in the electrolyte.

During CORR, the forming acetate ions cross the AEM separator. During long-term operation, the increasing acetate concentration decreases the anolyte pH, eventually resulting in an acetate buffer (pH between 4 and 6). This should lead to the stabilization of Ir (similar to the forming carbonate buffer during CO2RR^3^). Therefore, to directly study the effect of the anolyte pH, we repeated the electrolysis experiments employing 0.5 M acetate buffer as anolyte (pH ≈ 4.7, Figure 1). Higher anolyte pH resulted in a much slower voltage stabilization, though the overall voltage trends remained similar to the alkaline anolyte experiments. The change in process selectivity, however, followed an identical path, resulting in almost no CORR product formation after 2 h of electrolysis employing 0.5 M acetate buffer as anolyte. Again, the color of the anolyte changed during the measurement, but interestingly, to dark blue instead of purple.

A monotonous Ir concentration increase was found in the anolyte in this case as well, although in a lower amount (ca. 50% of what was measured with KOH anolyte).

Similar results were found (in terms of voltage profile and selectivity) when the cathodic CO feed was replaced with Ar (compared to when applying a KOH anolyte), except that the amount of Ir in the anolyte was notably lower (Figure 1). This could be due to the thermodynamic instability of Ir in basic solutions, through the formation of soluble IrO_4_^2–^.^34^ Nevertheless, as shown by all our experiments, contrary to almost all previous literature reports (accusing thermodynamics being the only suspect), our findings imply that *CORR products (or CO itself) crossing over the AEM during electrolysis have a decisive role in determining Ir stability.*

Finally, the cathode gas feed was changed to CO_2_ (all the other cell components and experimental parameters are identical to the CORR case). As shown in Figure S2, n -propanol, ethanol, and acetate are also formed as liquid products regardless of the pH of the electrolyte (the largest portion of the charge is consumed by CO and C_2_H_4_ formation). Interestingly, only a slight decrease in CO2RR selectivity can be spotted over the course of the 2 h-long experiment, which is the major difference compared to the CO feed case. Moreover, the presence of Ir was not detected with EDX in the used cathode GDE (Figure S1), indicating that there is no notable Ir crossover and deposition (at least within the timeframe of this experiment). Ir dissolution increased notably compared to the cases when HER was employed as the cathode process (Figure S3), however, the amount of Ir dissolved falls behind the values determined for the CORR case (about 50% less). The lower amount of Ir in the electrolyte might be correlated with the more than two-times lower amount of ethanol (15% FE vs 6% FE toward ethanol formation after 10 min of electrolysis) when CO2RR is driven at the cathode (compared to the CORR case).

To achieve better control over the pH and the concentration of various CORR products (ethanol and acetate) in the electrolyte, further control measurements were performed in a membrane-separated microfluidic electrolyzer cell, with the same Ir black catalyst. The catholyte pH was maintained during these measurements (1 M KOH), while the anolyte pH and the quality and quantity of the organic additive (i.e., ethanol, acetate, and acetaldehyde) were systematically varied between 4 and 14 and 10–500 mM, respectively (Figures 2 and S4).

**Figure 2:**
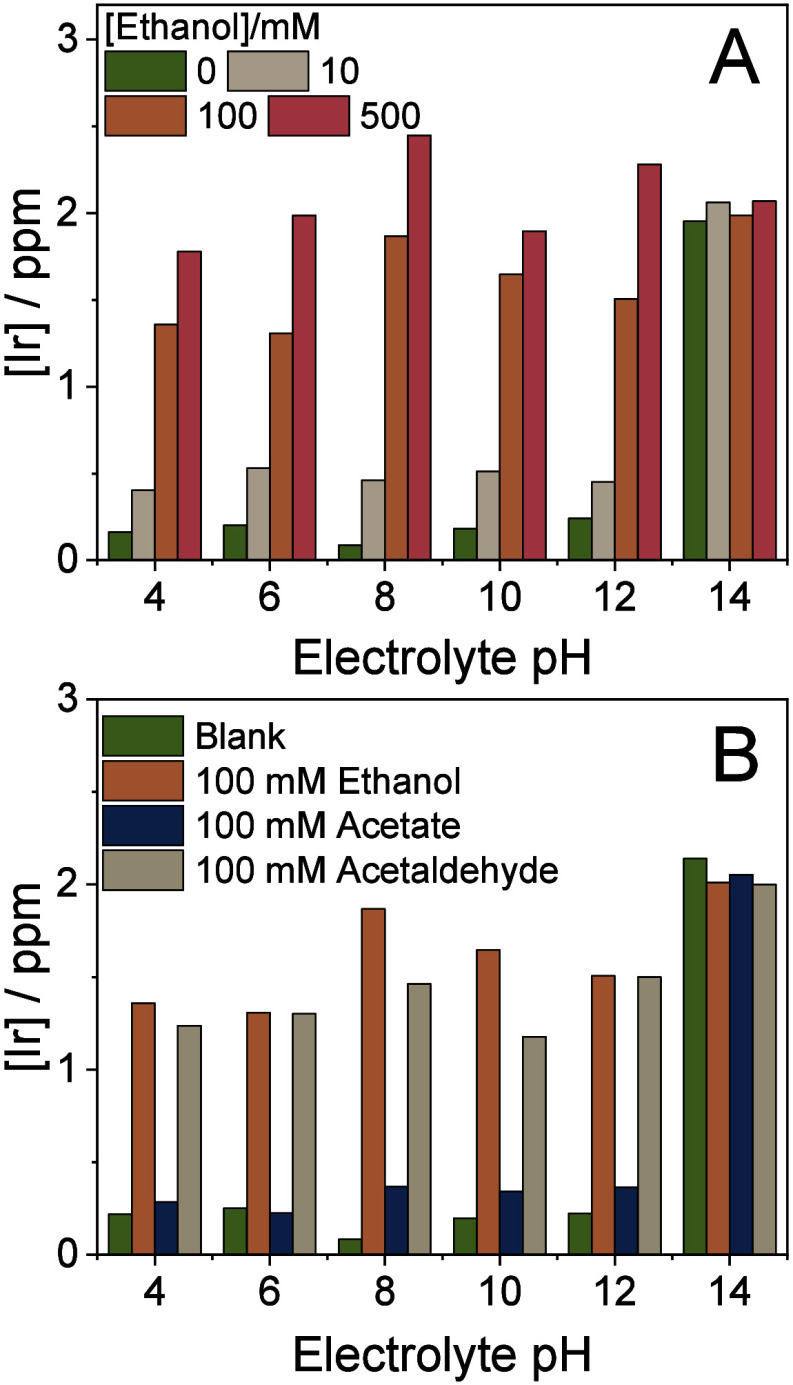
Dissolved amount of Ir during the constant current density measurements, presented in Figure S4. Measurements were performed using a membrane-separated microfluidic electrolyzer. The anolyte was analyzed by ex situ ICP-MS. (A) Ethanol-containing electrolytes, (B) comparison of ethanol, potassium acetate, and acetaldehyde-containing electrolytes.

In the absence of organics, the amount of dissolved Ir is the highest at pH = 14 (Figure 2), as implied by thermodynamics (i.e., Pourbaix diagram) and previous literature data.^17^ If the pH is decreased even to pH = 12, the rate of Ir dissolution decreases by an order of magnitude. Further decreasing the pH, the dissolution rate reaches a minimum at near neutral pH, and increases below this, in accordance with former studies.^17^ This trend drastically changes upon the addition of a small amount (10 mM) of ethanol to the electrolyte; Ir dissolution is 2–2.5 times higher than what was measured for the blank electrolytes at pH < 14. Furthermore, the Ir dissolution rate scales with ethanol concentration. At 500 mM ethanol concentration and near-neutral pH, the Ir dissolution rate exceeds that measured in the absence of organic additives at pH = 14! Recent research suggests that acetaldehyde, a partially oxidized intermediate of ethanol, may also reduce Ir stability.^28^ By introducing 100 mM acetaldehyde into the electrolyte, we observed a destabilization effect similar in magnitude to that of ethanol. This finding corroborates our hypothesis that the ethanol oxidation pathway is the principal driver of iridium degradation in this system. In contrast to ethanol and acetaldehyde, the addition of potassium acetate only slightly altered the dissolution characteristics, leading to the conclusion that ethanol is the species dominantly responsible for the destabilization of Ir and its subsequent dissolution from the catalyst layer.

To provoke the same destabilization effect while driving HER at the cathode in a zero-gap electrolyzer cell, 1.0 M ethanol was added to the anolyte (either 0.5 M KOH or 0.5 M pH ≈ 4.7 acetate buffer solution), while Ar was fed to the cathode (Figure S5 **)**. The addition of ethanol either to KOH or the acetate buffer caused a 4 times increase in the Ir concentration in the anolyte after 2 h of electrolysis. In contrast, only a minor increase in the case of the KOH anolyte can be witnessed. Notably, this ethanol concentration is much higher compared to that formed during our CORR experiments (compared to the KOH+CO case presented in Figure 1, ≈6 mM and ≈16 mM for in situ generated ethanol and acetate in the first 10 min of the electrolysis experiment, respectively). This indicates that the *local chemical environment* (most importantly, alcohol/aldehyde concentration and pH) plays a primary role in altering Ir stability.

### Identification of the Dissolved Iridium Species

In the case of the CORR experiments performed in the zero-gap setup, the gradual coloration of the transparent anolyte to dark blue or purple was observed. Besides Ir, Fe and Ti (metallic cell constituents) concentration was also monitored by ICP-MS during these experiments, but the concentration of the latter two metals remained below the detection limit.

While ICP-MS can precisely determine metal concentrations, it tells nothing about the chemical form in which Ir is present in the electrolyte. Therefore, it was first probed by recording UV–vis spectra for all the anolyte samples after the CORR electrolysis (Figure 3A). These were compared to UV–vis data collected for several reference solutions, assuming the formation of iridium-hydroxo/acetate coordination compounds (further information and reference spectra are presented in the SI - Figure S6).^35^ Although the formation of Ir-acetate complex has been proposed to be one of the main reasons behind increased Ir dissolution in the presence of primary alcohols,^28^ our results did not confirm this notion. According to former results, the spectra presented in Figure 3A are identical to the ones recorded for d = 1–3 nm, OH^–^, and H_2_O-capped IrO_2_ nanoparticles (band assignations are located in the SI, Table S1).^36–38^ These are typically synthesized by hydrolyzing a precursor salt (Ir^3+^ or Ir^4+^) in a strong alkaline medium forming an Ir­[(OH)_6_]^2–^ complex with an absorption maximum at around 310–330 nm. At suitably high concentration, a spontaneous condensation process occurs, resulting in OH^–^-capped IrO_2_ nanoparticles with a broad absorption centered at 560–580 nm.^36–38^ The nature of capping (i.e., OH^–^, or H_2_O-capped), depends on the pH of the electrolyte.

**Figure 3:**
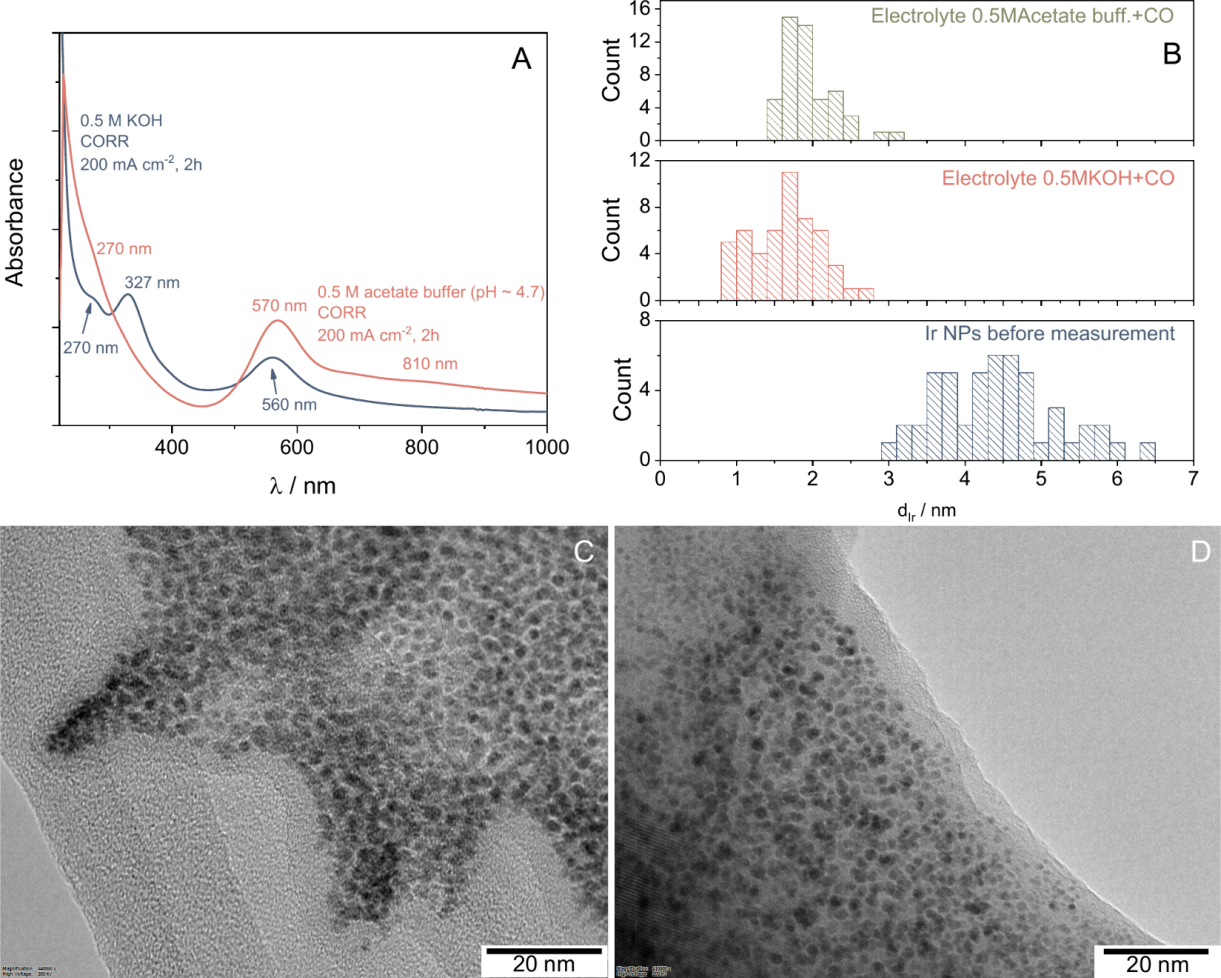
(A) UV–vis spectra recorded for the anolyte samples taken after the 2-h-long CORR electrolysis experiments presented in Figure 1. (B) Size distribution histograms of the Ir nanoparticles derived from TEM images presented in (C) and (D) and in Figure S7. Histograms were constructed by analyzing 50 individual particles on each image. TEM images recorded for the samples taken from (C) pH ≈ 4.7 acetate buffer + CO and for the (D) KOH+CO anolytes after the CORR experiment.

When the bulk electrolyte pH is lower (e.g., in the acetate buffer formed during CORR), the absorption band attributed to Ir­[(OH)_6_]^2–^ disappears. The different degrees of protonation (OH^–^ vs H_2_O-capped NPs), the different mean sizes of the NPs, and the higher IrO_2_ concentration under acidic conditions together are responsible for the difference in the observed color (dark blue vs purple) of the samples.

In parallel with recording the spectra, a small portion of each liquid sample was cast and dried on Lacey grids and TEM images were captured in a subsequent step (Figure 3 C and D, morphology of the pristine Ir black catalyst before electrolysis shown in Figure S7 **)**. To our surprise, both samples were full of evenly distributed small nanoparticles regardless of the applied electrolyte.

The morphology and size of these considerably differ from the bare Ir black (Figure 3B). The mean Ir nanoparticle diameter is around 4.5 nm in the case of the as-is sample, having a relatively broad size distribution (between 3 and 6.5 nm). Contrastingly, both postelectrolysis samples consisted of notably smaller particles (around 2 nm) with a narrower size distribution. These nanoparticles were identified as Ir/IrO_2_ based on electron diffraction and Fourier transform TEM results (see Figure S8 and Table S2). The notably decreased size of Ir after electrolysis suggests that these particles do not simply originate from the detachment of the anode catalyst layer, but chemical changes occur as well.

To glean insights into the oxidation state of the dissolved Ir species, liquid electrolyte samples (KOH+CO, and pH ≈ 4.7 acetate buffer+CO) were cast and dried on a glassy carbon plate, and XPS spectra were collected (Figure S9 and Table S3). The metallic nature of iridium oxide was accounted for by employing asymmetric line shapes (Doniach-Sunjic (DS)) together with satellite peaks during XPS fitting of the Ir 4f core-level region.^39,40^ In the case of both samples, apart from metallic iridium the presence of amorphous iridium oxide can be observed (the simultaneous presence of (III) and (IV) oxidation states of iridium. However, the ratio of the three species differs considerably (see Table S3).

Besides the condensation mechanism due to the local pH elaborated above, an additional reason for nanoparticle formation could be that ethanol acts as a reducing agent. This allows the formation of Ir^0^ atoms, and by their nucleation and growth, Ir nanoparticles in a subsequent step. Since this process is base-catalyzed, it most likely occurs only in electrolytes with higher pH. The first example in the literature was the “polyol” process, employing ethylene glycol as the solvent, stabilizing and most importantly as the reducing agent.^41^ Since then, the same process has been demonstrated for various noble metals but employing ethanol instead of ethylene glycol.^42,43^ At the highest pH, this could be a competing process with the formation of IrO_2_ nanoparticles (NPs) via the condensation pathway (*vide supra*). This might also explain the lower absorbance detected for the IrO_2_ NPs in 0.5 M KOH via UV–vis (Figure 3A), and the observations made for the TEM data (presence of Ir and IrO_2_ in the sample).

### Monitoring of Ir Dissolution in Real-Time

To better understand the kinetics of the dissolution process both in the absence and presence of ethanol and acetate, online ICP-MS measurements were performed.^44,45^ (Figures 4A and S10). Ir dissolution rates were the highest at the highest studied electrolyte pH (pH = 13). Depending on the applied potential and the electrolyte pH, there are two separate dissolution mechanisms that must be considered. At lower potentials, the formation of Ir^3+^ is followed by the rapid surface passivation, which is therefore a transient process. At higher potentials (typically under OER conditions), IrO_4_^2–^ can form, leading to the steady dissolution of the catalyst (a more detailed discussion is provided in the SI). The latter is the favored process under highly alkaline conditions.^17^ The introduction of 100 mM ethanol only slightly modified the dissolution characteristics at high pH (similar to the ex situ ICP-MS data presented in Figure 2). Furthermore, the onset potential of anodic dissolution was also not affected by the presence of ethanol (Table S4); it was 0.95 V_RHE_ in both cases, which is in line with former studies.^17,34,46^

**Figure 4:**
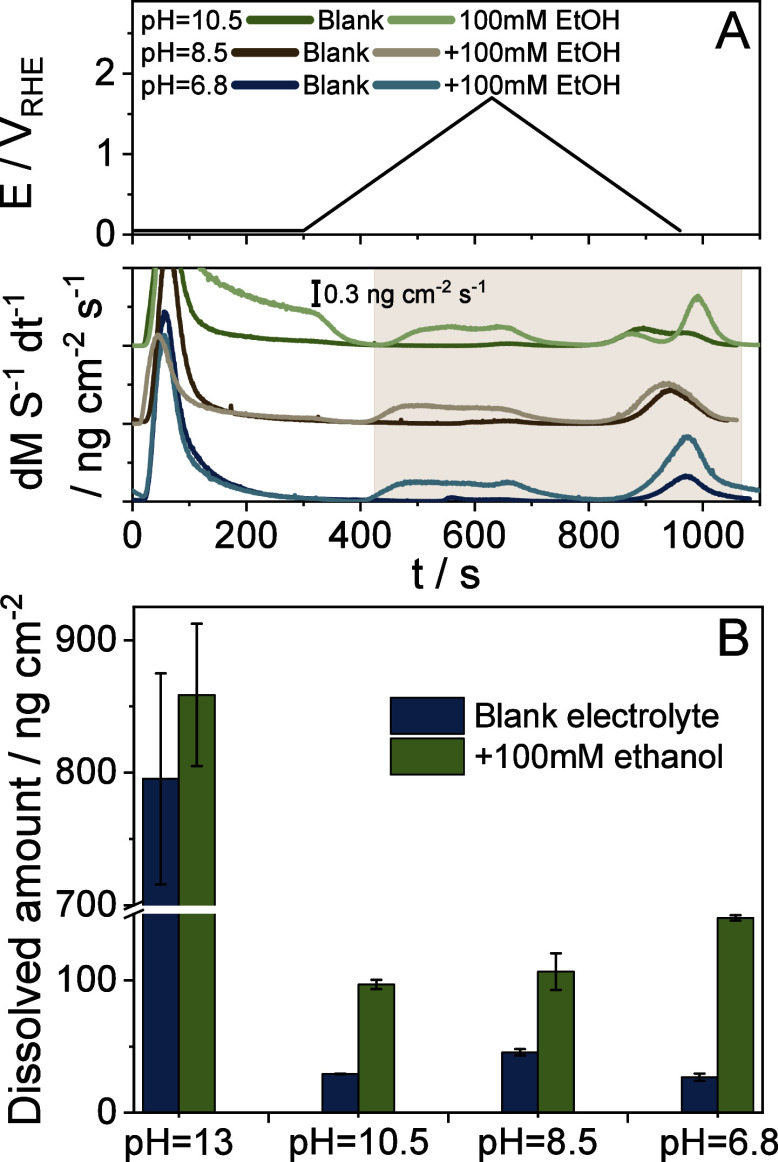
(A) Ir dissolution rates at different pH quantified by online ICP-MS recorded while performing the following electrochemical protocol: potentiostatic hold at E = 0.05 V_RHE_ for 5 min, followed by a cyclic voltammogram between 0.05 V_RHE_ – 1.6 V_RHE_ applying a 5 mV s^–1^ scan rate. (B) Dissolved amount of Ir calculated by integrating the dissolution rates presented in (A) and in Figure S10. The integrated area is highlighted with a light brown background in (A). Error bars were calculated from at least two measurements, each performed on a pristine sample.

A notable difference can be seen when the pH is lower than 13. In the ethanol-free electrolytes, Ir dissolution rate decreases greatly. However, at pH < 13, a striking increase in Ir dissolution rates was spotted upon the addition of ethanol to the electrolytes. This increase happened in parallel with the widening of the anodic and the first cathodic dissolution peak, resulting in a significant shift (between 500 and 700 mV!) in the onset potential of Ir dissolution to less positive potentials. Integration of the dissolution peaks yields the amount of Ir dissolved in the given potential window (see the total amounts in Figure 4B). The amount of dissolved Ir is the highest at pH = 13 (6 to 8 times higher compared to the lower pHs). Second, if pH < 13, Ir dissolution increases considerably if the electrolyte contains ethanol (5–6 times higher Ir content compared to the blank electrolyte), and the dissolved Ir concentration seems to be independent of the electrolyte pH.

Based on these results, our hypothesis is the following: ethanol interacts with the Ir catalyst surface, influencing its passivation, hence its stability. Only a handful of studies have been published on the stability-influencing aspects of electrocatalytic alcohol oxidation. Thorough studies were published on methanol or isopropanol oxidation on PtRu electrocatalyst,^24,25^ and on the stability of Ir and IrO_*x*_ in the presence of ethanol and acetaldehyde in mildly basic to strongly acidic electrolytes.^28,29^ In strong acidic media, Ir oxidation under high potentials (i.e., OER conditions) shares common intermediates with the primary alcohol oxidation process (IrO_3_). This could facilitate the formation of the soluble, IrO_4_^2–^ intermediate. Therefore, the presence of such organic species could catalyze Ir dissolution considerably even at low potentials.^29^ In our case, this is proven by the largely decreased onset potential of Ir dissolution in the presence of ethanol (Table S4).

To better mimic the conditions present during the electrolysis experiments, galvanostatic measurements were performed at j = 10 mA cm^–2^ (Figure S11). Conclusions regarding dissolution characteristics are similar to what was made for the potentiodynamic data. A clear dissolution peak can be identified in the presence of ethanol, the Ir signal does not return to its baseline, but seems to be stabilized at a lower value, just like in the pH = 13 case (where Ir is not stable and constantly dissolves as IrO_4_^2–^).^34^

## Conclusions and Outlook

Based on our results, the mechanism of Ir dissolution can be summarized as follows (visualized in Scheme 1):

1. Liquid-phase products forming during CO and CO_2_ electrolysis (most notably ethanol and acetate) cross over the AEM separator from the cathode side to the anode side.
2. Electrocatalytic oxidation of ethanol and its partially oxidized intermediate, acetaldehyde occurs on Ir (competing with the OER), in conjunction with the surface oxidation of Ir. As evidenced in previous studies,^47–49^ oxygenated surface species are consumed by the alcohol oxidation reaction, preventing the formation of a continuous passivating IrO_*x*_ layer. Additionally, the alcohol oxidation process occurs via the IrO_3_ intermediate that could facilitate the formation of the soluble IrO_4_^2–^ intermediate.^29^
3. Due to the acidified local pH (OH^–^ consumption during OER and the organic molecule oxidation processes), dissolved Ir species agglomerate to small (d = 1–3 nm) nanoparticles causing the coloration of the anolyte. At strong alkaline pH (typical for CORR) and in the presence of ethanol, Ir NPs might also form via the base-catalyzed reduction of the dissolved Ir species. Finally, the detachment of smaller Ir grains cannot be excluded, upon the dissolution of Ir from the grain boundaries of the bare NPs.

**Scheme 1:**
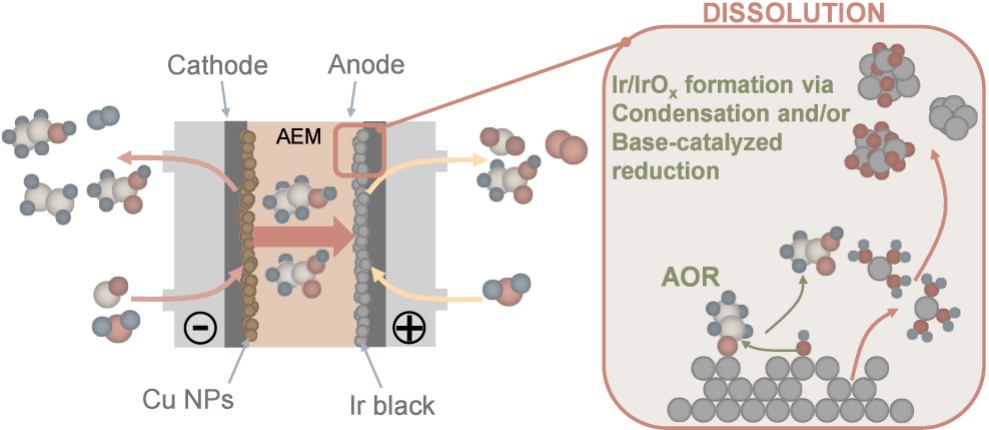
Degradation Mechanism of the Ir Anode Catalyst Layer under OER Conditions^a^

a If CORR is the cathode process, the CO liquid product crossover is followed by the partial electrocatalytic oxidation of ethanol and its oxidized intermediate, acetaldehyde present in the anolyte, which starts in conjunction with the oxidation of the catalyst surface. Alcohol/aldehyde oxidation consumes oxygenated surface species, leading to increased Ir dissolution. Due to local pH effects/the role of ethanol as a reducing agent leads to the formation of Ir/IrO_*x*_ nanoparticles, altering the color of the anolyte.

Catalyst loss is just one consequence of the dissolution process; the dissolved metal cations can cross over the AEM and deposit on the cathode catalyst layer. This can irreversibly alter the selectivity of the cathode catalyst (shifting from CORR to HER) and terminating the long-term operation of the electrolyzer cell.^3,15^ Ir dissolution can be minimized if liquid product crossover from the cathode side is mitigated. Additionally, if strong alkaline electrolytes are used (e.g., CO electrolysis) Ir should be replaced with materials that are stable under these conditions.

Our results underline the necessity of studying the effects of small organic molecules typically forming during CO2RR/CORR on the stability of the anode catalyst, which has been a neglected topic so far.SI; Careful identification and mitigation of such factors is an essential ingredient in achieving high performance and stability on a longer time scale.

## Supplementary Material



## Notes

Experimental part. Scheme S1, Figures S1–S11, Tables S1–S4. Additional materials characterization and electrochemical data; SEM-EDX, TEM, XPS, galvanostatic electrolysis results performed both in the microfluidic- and zero-gap cell setups, Ir dissolution during electrolysis determined by ex-situ ICP-MS, UV–vis spectra recorded in reference electrolytes, online ICP-MS data employing cyclic voltammetry and galvanostatic hold protocols and tracking Ir dissolution in real time, additional CORR and CO2RR selectivity data. (PDF)
