# Supplementary material for: Microenvironment Matters: Destabilization of Iridium Anode Catalyst by CO Reduction Products
Source: J Am Chem Soc. 2026 Feb 19;148(8):9006–13. doi: 10.1021/jacs.5c22283 (PMC12964409; doi:10.1021/jacs.5c22283)
Supplement: Supplementary file 1 [file ja5c22283_si_001.pdf]

# SUPPORTING INFORMATION

## Microenvironment Matters: Destabilization of Iridium Anode Catalyst by CO Reduction Products

Attila Kormányos,[1, ‡,*] Mohd Monis Ayyub,[1,‡] Bence Kutus,[2] Monaza Rashid,[1] Tatiana Priamushko,[3] Gergely F. Samu,[2] Serhiy Cherevko,[3] Balázs Endrődi,[1,4] Csaba Janáky[1,5*]

[1]Department of Physical Chemistry and Materials Science, University of Szeged, Rerrich square 1, Szeged H-6720, Hungary

[2]Department of Molecular and Analytical Chemistry, University of Szeged, Dóm square 7-8, Szeged H-6720, Hungary

[3]Forschungszentrum Jülich GmbH, Helmholtz-Institute Erlangen-Nürnberg for Renewable Energy (IET-2), Cauerstraße 1, 91058 Erlangen, Germany

[4]MTA-SZTE Lendület "Momentum" Applied Electrochemistry Research Group, University of Szeged, Rerrich square. 1, H-6720 Szeged, Hungary

[5]Interdisciplinary Excellence Center, University of Szeged, Rerrich square 1, Szeged H-6720, Hungary

[‡]These authors contributed equally.

*e-mail: janaky@chem.u-szeged.hu, kormanyos.attila@szte.hu

EXPERIMENTAL SECTION

**Chemicals.** Potassium hydroxide (KOH, VWR), isopropanol (IPA, VWR), copper nanoparticles (Cu NPs, $d_{avg}$ = 25 nm, Sigma-Aldrich), iridium black (Ir, high surface area, FuelCellStore) were of analytical grade and used without further purification. Capstone ST-110 (CST, 25 wt% aqueous dispersion) and Nafion (10 wt% aqueous dispersion) were used as the ionomers purchased from Chemours and Fuel Cell Store, respectively. A 4.7 purity CO (Messer) and 4.7 purity Ar (Messer) were used. All electrolyte solutions were prepared using MilliQ water (Millipore Direct Q3-UV, 18.2 MΩ cm).

**Catalyst layer preparation.** All gas diffusion/porous transport layer electrodes used in this study were prepared by spray-coating. For this, a handheld airbrush (Alder AD-320) and compressed air ($p$ = 0.6 barg) were used. All substrates were placed on a hotplate heated to $T$ = 100 °C during spray-coating.

In the case of the *cathode* catalyst layers, depending on the cell, two types of carbon paper gas diffusion layers (GDL) were used: microfluidic cell - Freudenberg H23C6 GDL, zero-gap cell – Sigracet 28BC GDL. Copper NPs were dispersed in 1:1 $H_2O$:isopropanol mixture with 5 wt% Capstone ST110 binder. The dispersion was sonicated for 20 minutes. The catalyst loading was maintained at 1 mg $cm^{-2}$.

Ir black was used as the *anode* catalyst. The anode catalyst ink was prepared by mixing Ir black NPs with 1:1 isopropanol:$H_2O$ mixture with adding 15 wt% Nafion ionomer and sonicated for 20 minutes prior to use. The Ir black dispersion was spray-coated either on a Freudenberg H23C6 GDL (microfluidic cell) or on a Ti frit porous transport layer (PTL, zero-gap electrolyzer cell). The catalyst loading was 1 mg $cm^{-2}$.

**Physical and morphological characterization.** Morphological features of the Ir NPs found in the various electrolytes after the CORR experiments were explored with transmission electron microscopy (TEM) using a FEI TECNAI G2 20 X-Twin TEM with an accelerating voltage of 200 kV. All samples were prepared on a lacey carbon support by drop-casting 1 µl of electrolyte and letting it dry under ambient conditions.

Crystal structure of the Ir black NPs was studied with X-ray diffraction (XRD) using a Rigaku MiniFlex II instrument with a Cu Kα (λ = 1.5418 Å) X-ray source. Operating conditions were 30 kV, 15 mA in the 10°- 80° 2Θ range, applying a scan speed of 1.0° $min^{-1}$.

UV-vis spectra were recorded for samples drawn from each electrolyte at the end of the CORR electrolysis experiments employing the zero-gap electrolyzer cell using a Shimadzu UV-3600 Plus spectrophotometer in between 200 and 1000 nm in absorbance mode. All spectra were recorded using a quartz cuvette ($l$ = 1 cm) without diluting the samples.

To examine the composition of the drop-cast films, X-ray photoelectron spectroscopy (XPS) measurements were performed with a SPECS instrument equipped with a PHOIBOS 150 MCD 9 hemispherical analyzer. The analyzer was operated in fixed transmission mode with 40 eV pass energy for acquiring survey scans. For the high-resolution scans 20 eV pass energy was used. Al $K_a$ radiation (h v = 1486.6 eV) was used as an excitation source and operated at 150 W power. Multiple scans were averaged to get a single high-resolution spectrum. Charge compensation was carried

out with an electron flood gun. Charge referencing was performed for the adventitious carbon C 1s peak (284.8 eV). For spectrum evaluation, CasaXPS commercial software package was used.

**Electrochemical experiments.** All electrochemical measurements were performed using a Biologic VMP-300 potentiostat. Gas-phase CORR products were quantified by a Shimadzu Nexis GC-2030 gas chromatograph, equipped with a TCD detector and an automated 6-way valve injection system. Liquid products were analyzed by nuclear magnetic resonance spectroscopy using a Bruker AV-III-500-HD NMR. NMR calibration was carried out for the compounds of ethanol, acetate and n-propanol in an environment mimicking the one employed in the electrolysis experiments.

Electrochemical measurements were performed in two distinctly different cell configurations (**Scheme S1**). *Configuration I* employs a membrane-separated microfluidic electrolyzer cell. Here the effect of liquid CORR products were investigated separately by adding them directly in the applied anolyte solution, while no CO was fed to the cell, hence HER was the cathode process. The catholyte and anolyte channels were machined from 2 mm thick PEEK sheets (2 x 0.5 cm window size, with 1 mm in diameter holes on both ends to allow electrolyte flow). The active geometric area of the cathode and anode catalyst layers is 1 $cm^2$. 3 mm-deep cavities with two M5 threaded inlet/outlet ports were formed on both stainless-steel current collectors acting as gas-flow channels. A 40 µm thick PiperION AEM (activated in 1 M KOH) was sandwiched between the electrolyte channels. The sealing of the cell was ensured by thin PTFE gaskets (d = 100 µm – anode side, d = 200 µm – cathode side). 1 M KOH was employed as catholyte. The anolyte was either 1 M KOH or 1 M $KNO_3$, of which pH was set by the addition of concentrated KOH or $HNO_3$ along with the addition of either ethanol or potassium acetate (c = 10 – 500 mM). The catholyte was flown through the cell in a single-pass mode, while V = 10 $cm^3$ anolyte was recirculated throughout the duration of the measurement. Both electrolyte flow rates were set to 1 $cm^3$ $min^{-1}$ and electrolyte flow was maintained by a two-channel peristaltic pump (Ismatec). Room temperature Ar was fed to the cathode compartment of the cell with 12 $cm^3$ $min^{-1}$ flow rate. Gas flow was controlled by a Bronkhorst EL-FLOW Select F-201CV mass flow controller. A galvanostatic protocol was used applying a current density of j=100 mA $cm^{-2}$ for 10 min.

*Configuration II* is an 8 $cm^2$ active geometric area zero-gap type cell designed and manufactured in-house (**Scheme S1**). The cathode and anode compartment of the electrolyzer is separated by the same, 40 µm-thick PiperION AEM as in the case of the microfluidic cell. A 230 µm PTFE spacer was placed in the cathode compartment. Room-temperature CO, $CO_2$ and Ar is introduced in the cell without humidification via the back of the cathode current collector employing a gas flow pattern with a flow rate of 100 $cm^3$ $min^{-1}$. Gas flow was controlled using a Bronkhorst EL-FLOW Select F-201CV mass flow controller. V = 40 $cm^3$ (to amplify any effect related to the change in the anolyte composition) 0.5 M KOH was recirculated through the anode compartment of the cell with a flow rate of 60 $cm^3$ $min^{-1}$ using a peristaltic pump. Either ethanol or potassium acetate was added to the KOH-containing electrolyte, along with performing control experiments in a pH = 6 acetate buffer with or without ethanol or potassium acetate. The electrochemical protocol consisted of a galvanostatic hold applying j = 200 mA $cm^{-2}$ current density for either two or three hours while periodically taking aliquots from the anolyte for further analysis (NMR and ICP-MS).

**On-line ICP-MS.** Dissolution of iridium during electrolysis was scrutinized by on-line ICP-MS in real-time. The setup consists of a three-electrode scanning flow cell that's outlet is directly connected to the inlet of the ICP-MS (Perkin Elmer Nexion 350X). A glassy carbon rod (SIGRADUR) was used as the counter and an Ag/AgCl/ 3M KCl electrode (Metrohm) as the reference electrode. The same Ir black suspension was used to fabricate the catalyst layers as in the case of the zero-gap, and microfluidic measurements. The suspension was drop-casted (0.2 µl, 4 mg Ir per catalyst spot) on a glassy carbon electrode (5x5 cm, SIGRADUR). This served as the working electrode after drying. All measured current densities are related to the geometric surface area of the given catalyst spot (measured by a laser scanning microscope – Keyence VK-X250). All potential values are reported against the reversible hydrogen electrode (RHE). Measurements were performed in 0.1 M KOH (pH = 13), 0.1 M $KNO_3$ (pH = 6.8), or 0.1 M $KNO_3$, which pH was set by the addition of 1 M KOH to either pH = 8.5 or 10.5. The electrolytes were used as-is or after the addition of 10 or 100 mM ethanol. Two electrochemical protocols were carried out. The first one consisted of a potentiostatic hold at E = 0.05 $V_{RHE}$ for 5 min followed by recording a CV between 0.05 $V_{RHE}$ and 1.6 $V_{RHE}$ applying 5 mV $s^{-1}$ scan rate.

The potential was held at open circuit for 5 minutes for the second protocol, which was followed by a galvanostatic hold applying j = 10 mA $cm^{-2}$ current density for 5 more minutes. A Gamry Reference 600 potentiostat was used to carry out all electrochemical protocols. The Ar-purged electrolyte flow rate was regulated by the peristaltic pump of the ICP-MS (MP2, Elemental Scientific) The average electrolyte flow rate was 3.46 ± 0.05 µl $s^{-1}$. ICP-MS was calibrated daily (or within a day when switching electrolyte) by a four-point calibration slope diluted from standard solutions (Merck Certipur Ir and Re). $^{187}$Re served as an internal standard, which was mixed with the sample flow via Y-connector then introduced in the ICP-MS through a nebulizer.

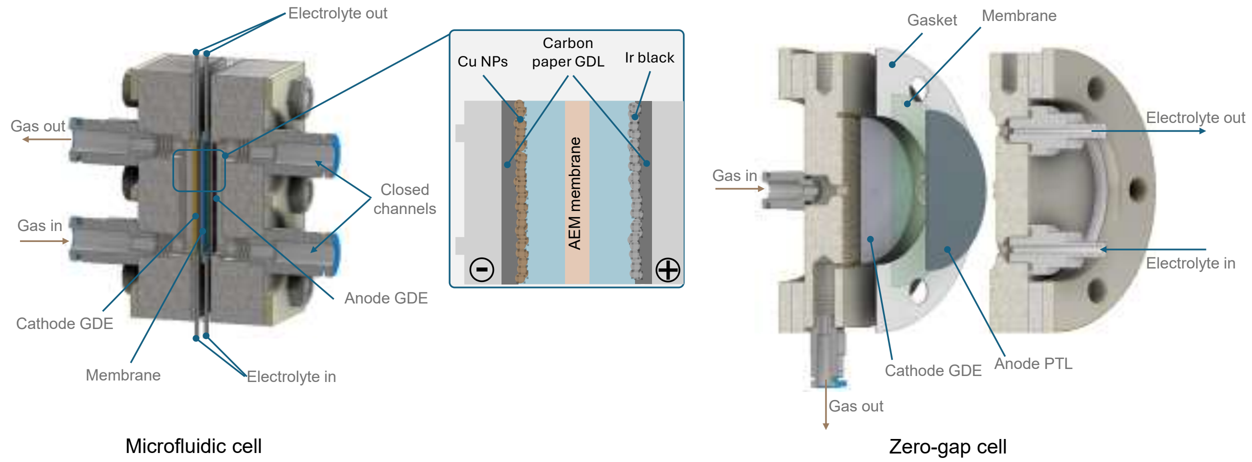


**Scheme S1.** Schematic representation of two of the continuous-flow electrolyzer cells employed in this study.

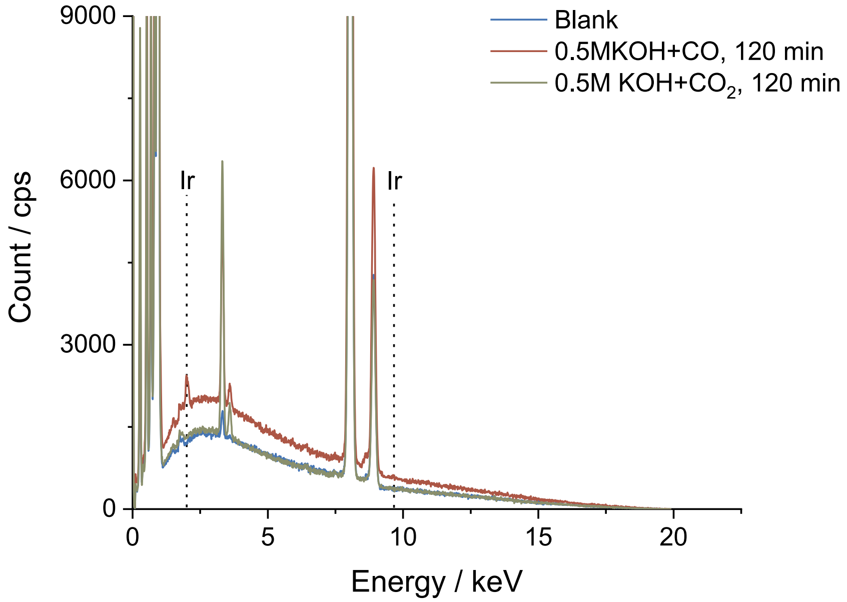


**Figure S1.** EDX spectra recorded for various cathode GDEs after performing a galvanostatic electrolysis protocol applying j = 200 mA cm$^{-2}$ for 120 min. “Blank” means that the cell was assembled as usual however, no electrolysis experiments were performed. Instead of that, the cell was disassembled, and the cathode GDE was examined further.

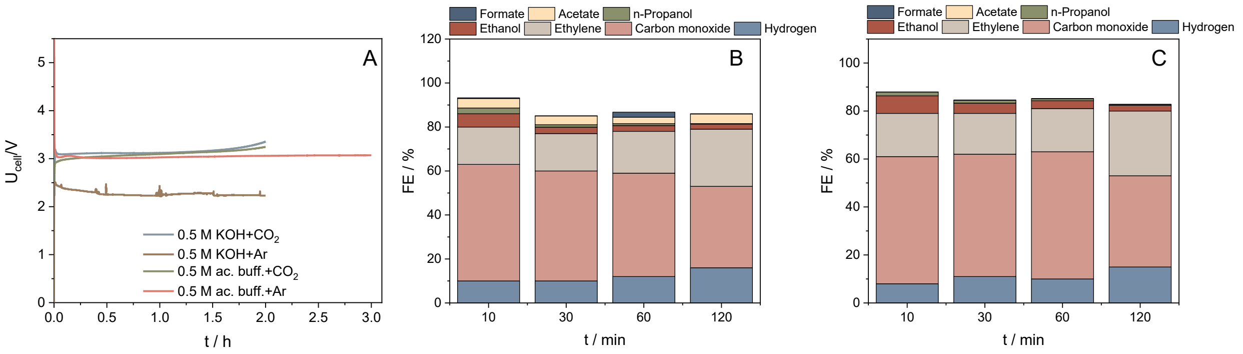


**Figure S2.** (A) Galvanostatic electrolysis results at j = 200 mA cm$^{-2}$ current density recorded in either 0.5 M KOH or 0.5 M acetate buffer (pH ≈ 4.7) anolyte (V = 40 cm$^3$, 60 cm$^3$ min$^{-1}$ flow rate). Non-humidified $CO_2$ or Ar was fed to the cathode side with a flow rate of 100 cm$^3$ min$^{-1}$. Product distribution for the (B) 0.5 M KOH+$CO_2$ or (C) 0.5 M acetate buffer+$CO_2$ case, monitored during the electrolysis measurement (products were collected from both the cathode and anode side of the cell).

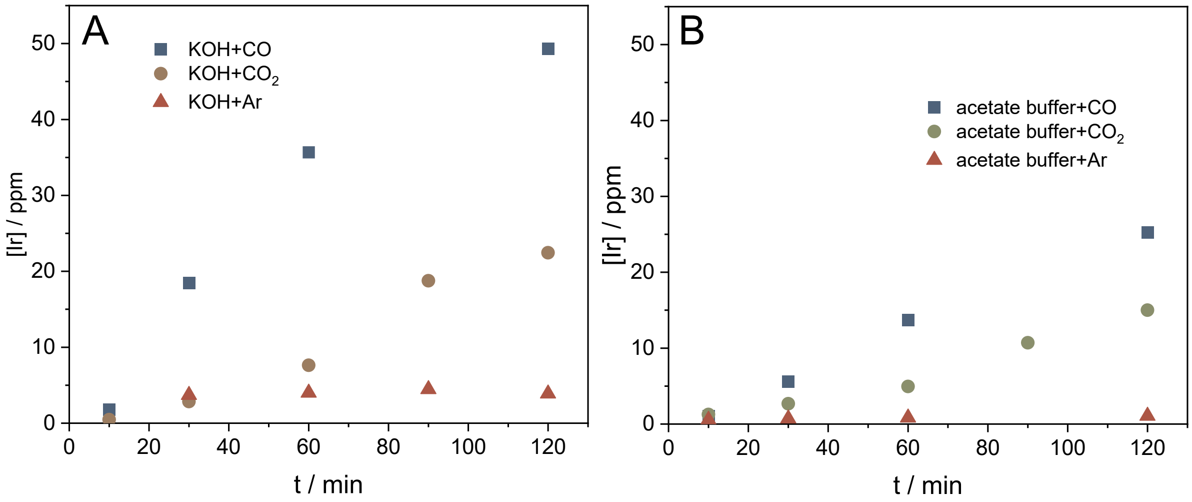


**Figure S3.** The amount of iridium dissolved in the liquid-phase aliquots taken during $CO_2$ or CO electrolysis experiments. The electrochemical protocol consisted of a galvanostatic hold employing j = 200 mA $cm^{-2}$ current density for 120 minutes. Either 0.5 M KOH or a 0.5 M, pH = 4.7 acetate buffer were employed as the anolyte (V = 40 $cm^3$, 60 $cm^3$ $min^{-1}$ flow rate). Non-humidified $CO_2$, CO or Ar was fed to the cathode side with a flow rate of 100 $cm^3$ $min^{-1}$. CORR and HER results are plotted on the Figure to serve as a reference.

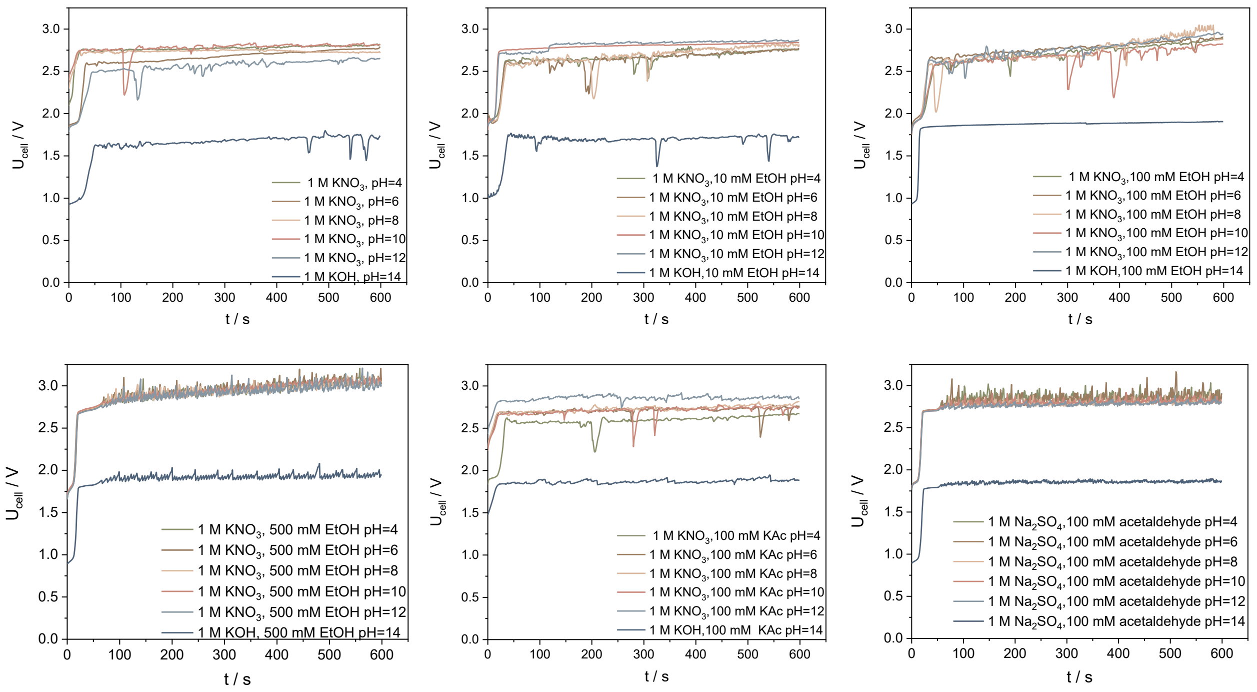


**Figure S4.** $U_{cell}$ vs. t curves applying j = 100 mA $cm^{-2}$ current density recorded in the pH-range 4-14. Ir+15 wt% Nafion was employed as the anode; and Cu nanoparticles+5 wt% Castone ST-110 as the cathode catalyst layers. Measurements were performed either in 1 M KOH or 1 M $KNO_3$ electrolyte solutions adding 0-500 mM ethanol or potassium acetate. pH was set by the addition of concentrated $HNO_3$ or KOH solutions. The cathode and anode compartment was separated by a 40 µm thick PiperION AEM membrane. 1 M KOH was used as catholyte in the case of all measurements. The electrolyte flow rate was kept 1 $cm^2$ $min^{-1}$.

According to the $U_{cell}$ vs. t curves, lowest cell voltage values were recorded at the highest pH (pH = 14, around 1.75 V), which then jumps to and stabilizes between 2.75-3.00 V when the electrolyte pH is reduced. The addition of organic molecules had no effect on this trend except when 500 mM ethanol was added to the anolyte (the cell voltage exceeds 3.00 V at lower than 14 pHs).

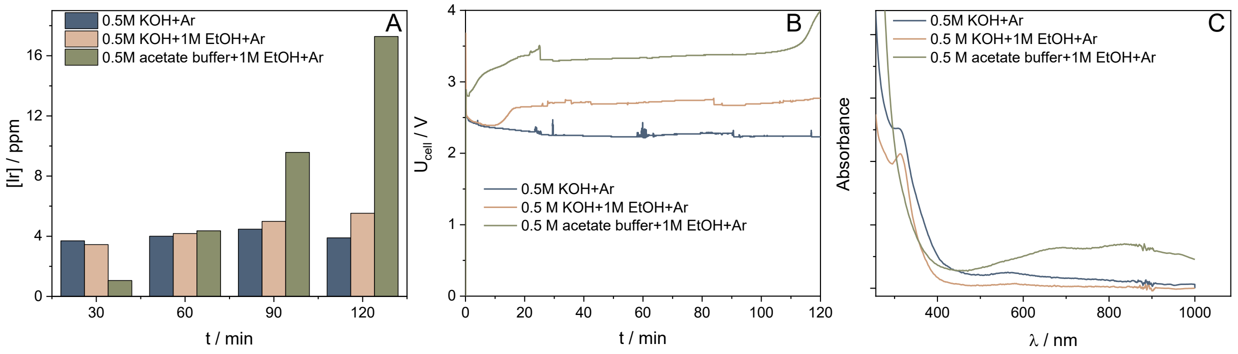


**Figure S5.** (A) The amount of iridium dissolved in the liquid-phase aliquots taken during the experiment presented in (B) and analyzed by ex-situ ICP-MS. (B) Galvanostatic electrolysis results employing j = 200 mA $cm^{-2}$ current density for two hours. Either 0.5 M KOH or a 0.5 M, pH ≈4.7 acetate buffer was employed as the anolyte (V = 40 $cm^3$, 60 $cm^3$ $min^{-1}$ flow rate) with or without the addition of 1 M ethanol. Non-humidified CO or Ar was fed to the cathode side with a flow rate of 100 $cm^3$ $min^{-1}$. (C) UV-vis spectra recorded for the anolytes after performing the electrolysis presented in (B).

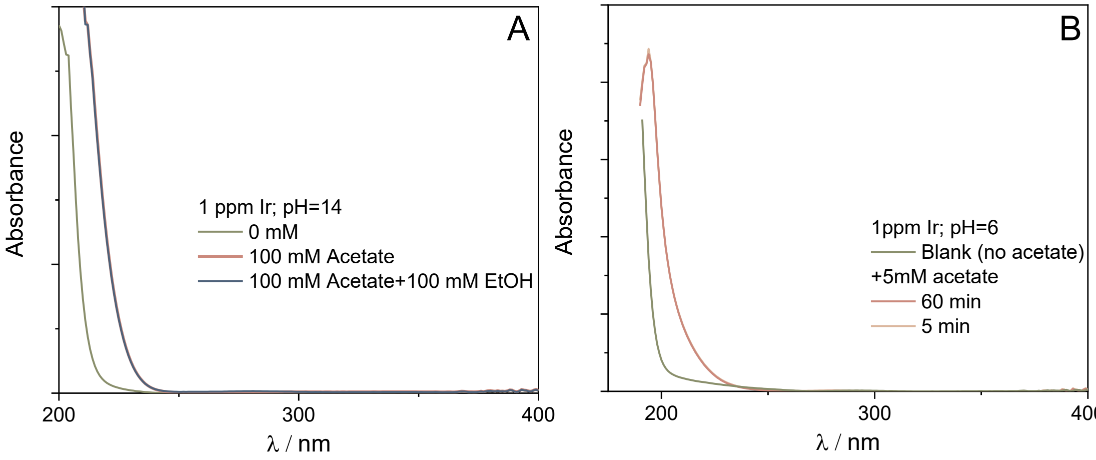


**Figure S6.** UV-vis spectra recorded for reference electrolytes. (A) 1 ppm Ir in a pH = 14 KOH solution. Potassium acetate, acetate and ethanol were systematically added to the blank solution in 100 mM concentration. (B) pH = 6 (set by cc. $HNO_3$), 1 ppm Ir concentration. 5 mM potassium acetate was added to the solution and change in the UV-vis spectra was followed for 60 min. For the sake of clarity, only spectra recorded at 5 and 60 minutes are presented besides the blank spectrum in the figure.

Supplementary notes for the data presented above:

- 1ppm $Ir^{3+}$ concentration was achieved by diluting a 1 g $dm^{-3}$ $Ir(NO_3)_3$ ICP standard (Merck Certipur) in MilliQ water. The pH was set by the addition of 5 M KOH or cc. $HNO_3$.
- A sharp absorption band appears upon the addition ethanol and acetate in the case of both sets of data, but there is no color change, and the spectra does not resemble the feaures appeared on the ones presented in Figure 3.
- The data presented in **Figure S6B** was performed by setting the pH of the solution to 6. Our assumption was that if an Ir complex forms at this pH, at least part of the OH- ions should be replaced by acetate in the complex. Based on the set of spectra presented, no such phenomenon can be identified.

**Table S1.** Assignation of bands appeared on the UV-vis spectra (presented in **Figure 3.**) recorded for electrolytes samples taken after performing a 3-hour long CORR electrolysis at j = 100 mA $cm^{-2}$ current density in either 0.5 M KOH or 0.5 M acetate buffer (pH ≈ 4.7) electrolyte solution.

| **λ / nm** | **Species** | **Reference** |
|---|---|---|
| **0.5 M KOH+CO** | | |
| 270 | | |
| 327 | $Ir[(OH)_6]^{2-}$ | [1–3] |
| 560 | $OH^-$-capped $IrO_2$ nanoparticles | |
| **0.5 M acetate buffer (pH ≈ 4.7)+CO** | | |
| 270 | | |
| 570 | $H_2O$-capped $IrO_2$ nanoparticles | [1–3] |

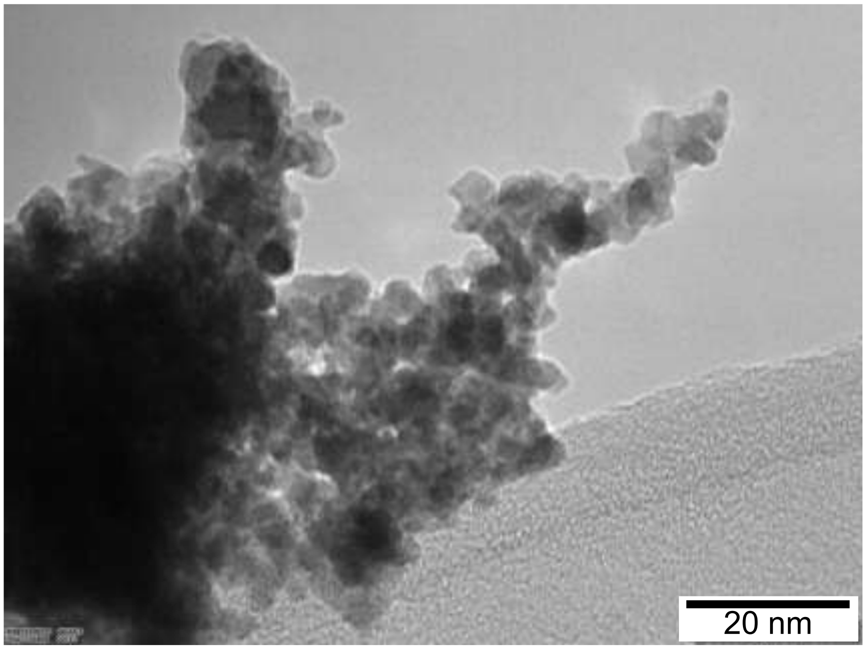


**Figure S7.** TEM image recorded for the Ir black nanoparticles used for the preparation of the anode catalyst layer.

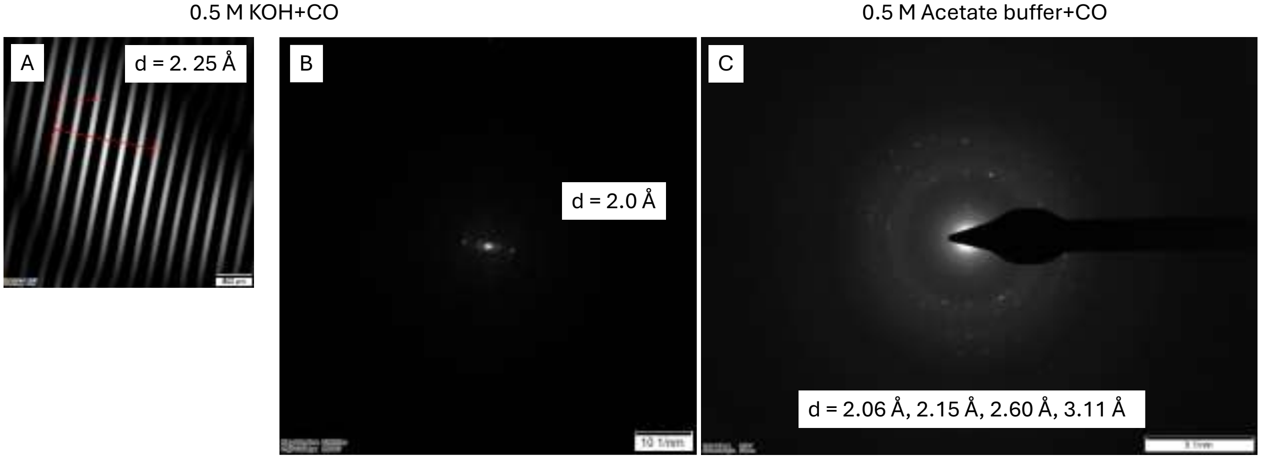


**Figure S8.** TEM and ED data used for the determination of the d-spacing values. Values corresponding to Ir are summarized in Table S2.

**Table S2.** d-spacing values determined from the TEM images and characteristic diffractions of Ir, KOH, and acetate.

| Material | 2Θ / ° | d / Å | Plane | d - derived from the TEM/ED data / Å | Reference |
|---|---|---|---|---|---|
| Ir | 40.58 | 2.22 | (111) | 2.25, 2.15 | 00-065-1686 POW-COD database |
| | 47.30 | 1.92 | (200) | 2.06 | |
| | 69.14 | 1.36 | (220) | - | |
| $IrO_2$ | 40 | 2.25 | (200) | 2.25, 2.30 | 2101854 POW-COD database |
| | 34.7 | 2.59 | (101) | 2.58 | |
| | 28 | 3.19 | (110) | 3.21, 3.24 | |

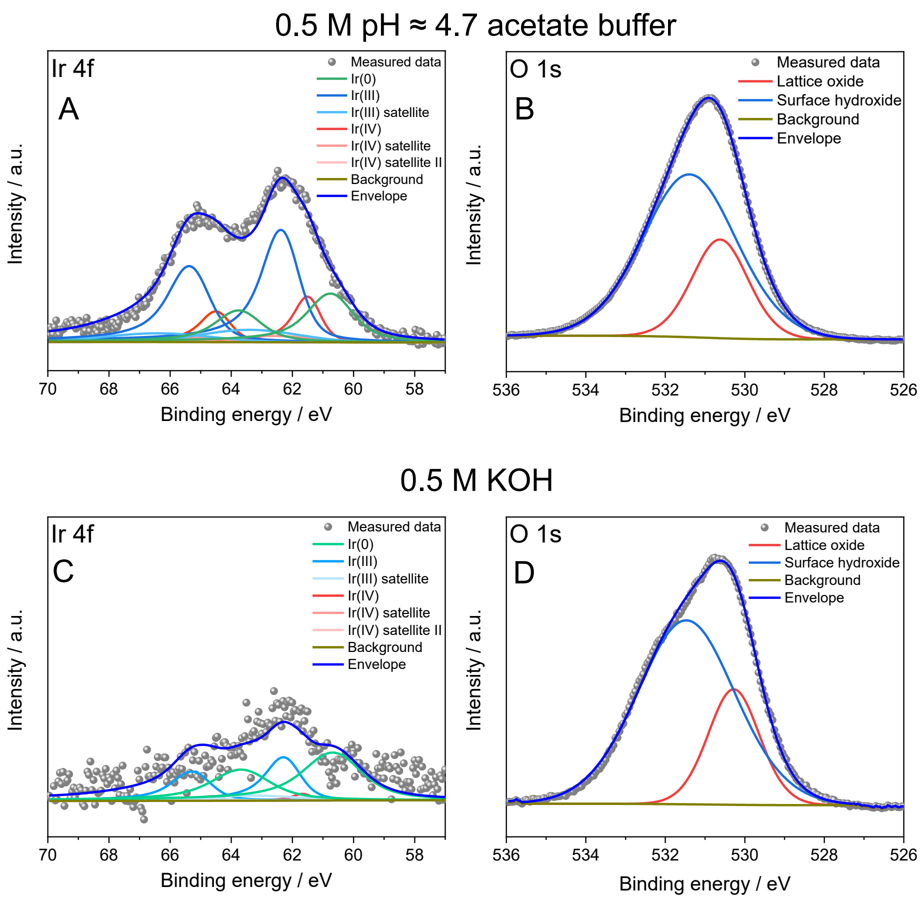


**Figure S9.** (A),(C) Ir 4f and (B),(D) O1s XPS spectra recorded for the anolyte samples after performing a three-hour-long electrolysis protocol in the zero-gap cell configuration applying j=200 mA $cm^{-2}$ current density. To prepare the XPS samples, 200 µl (in four 50 µl aliqots) electrolyte was drop-casted and dried on a 1 cm x1 cm GC plate polished and cleaned previously.

**Table S3.** Composition of the Ir/$IrO_x$ species derived from the XPS spectra presented in Figure S9.

| Composition | Ir(0) / at% | Ir(III) / at% | Ir(IV) / at% |
|---|---|---|---|
| 0.5M pH≈4.7 acetate buffer | 30 | 54 | 16 |
| 0.5 M KOH | 62 | 35 | 3 |

**Supplementary notes for the stability of Ir in alkaline media:**

Iridium dissolution characteristics:

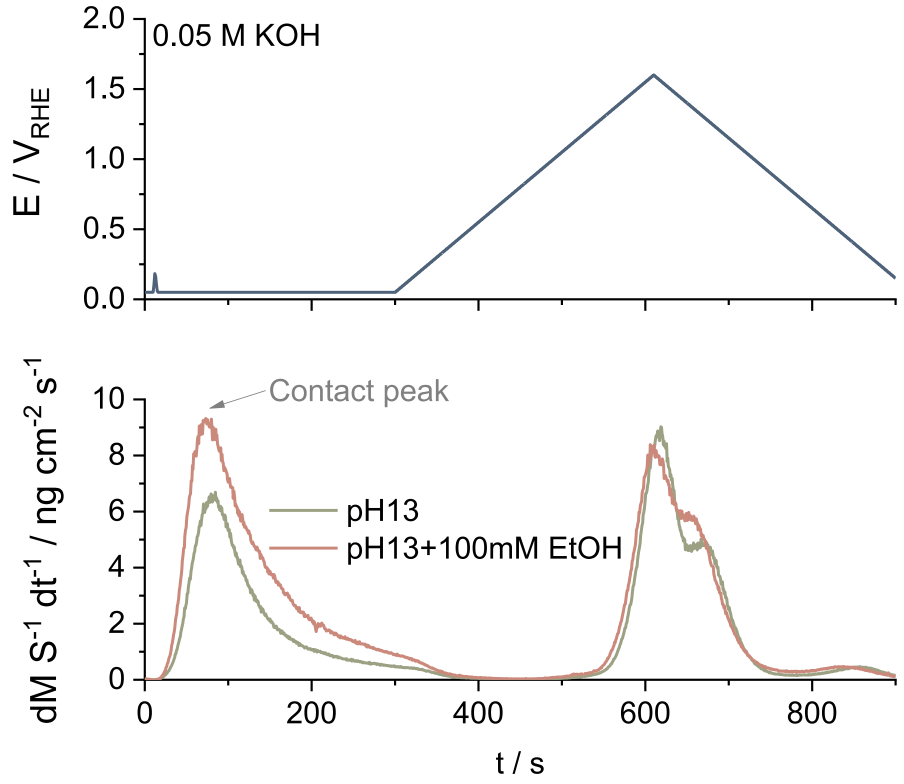


**Figure S10.** Stability of Ir in 0.05 M KOH under potentiodynamic conditions in 0.05 M KOH and in 0.05M KOH+100mM EtOH. The potential was scanned between 0.05 $V_{RHE}$ and 1.60 $V_{RHE}$ by applying a sweep rate of 5 mV $s^{-1}$. Ir dissolution in real-time was monitored by on-line ICP-MS.

In general, thermodynamic information along with detailed experimental data on the stability of noble metals under alkaline conditions is scarcely available.[4,5] Ir is no exception; most studies aiming understanding its corrosion were conducted in acidic electrolytes[6–8] due to Ir's practical relevance in PEM water electrolyzers and fuel cells.

The stability of Ir under electrocatalytic conditions can be scrutinized with on-line ICP-MS, which results for Ir are presented in **Figure S10**. The first peak, which is visible is the so-called 'contact peak' that emerges when the scanning-flow cell gets in to contact with the catalyst surface. This dissolution corresponds to the reduction of a native oxide layer inherently present at the catalyst surface when it is handled and stored under air.[9] Besides the contact peak, there are two main dissolution features that can be distincted. The first one appeared

during the forwards scan and is generally termed as ‘anodic dissolution’. The onset potential of Ir dissolution is at 0.95 $V_{RHE}$. This transient dissolution feature corresponds to the oxidation of the Ir catalyst surface and the formation of $IrO_{2,}$ as in the equation SE1 for acidic media.

$$Ir + 2H_2O \rightarrow IrO_2 + 4e^- \qquad \text{(SE1)}$$

As it has been described for Pt (111) single crystals,[10] after the oxidation of the topmost monolayer of the metal, the crystal lattice needs to be rearranged in a way to incorporate O atoms in the lattice. This occurs via a place exchange mechanism. This rearrangement occurs via the breakage of M-M bonds. Undercoordinated metal atoms at the catalyst surface are exposed to the electrolyte solution and they either be incorporated in the developing oxide layer or dissolve in the electrolyte. The determined onset potential of Ir dissolution is in good agreement with  the one that can be derived from thermodynamics.[11] Oxide formation continues until the whole catalyst surface is passivated. Under strong alkaline conditions, if the potential is further increased, $IrO_2$ is not stable. Hence, it forms soluble $IrO_4^{2-}$ species increasing further Ir concentration in the electrolyte.

To summarize, there are two competing Ir oxidation/dissolution processes depending on the pH of the electrolyte and the applied potential. Under OER conditions:[5]

pH < 7: transient dissolution is the main process driven dominantly by the formation of $Ir^{3+}$ cations. The dissolution process is rapidly quenched by the formation of a continuous $IrO_x$ layer protecting the electrode surface.

pH 7 – 11: The dissolution mechanism is determined by the competition between $Ir^{3+}$ formation (transient) and further oxidation of $IrO_2$ to $IrO_3$ and the formation of soluble $IrO_4^{2-}$ species (continuous dissolution).

pH > 11: Ir dissolution is dominated by $IrO_4^{2-}$ formation hence the considerably higher dissolution rates recorded for Ir in our study when the electrolyte pH is around 13.

There is one dissolution feature that can be spotted on the reverse scan termed as ‘cathodic dissolution’. This corresponds to the reduction of the previously formed oxide, which occurs in parallel with the dissolution of the undercoordinated metal sites [4,10] Out of these two features, the second one has an onset centered at 0.6$V_{RHE}$ signaling that the reduction of $IrO_x$ is kinetically hindered in the alkaline electrolyte.[12]

**Table S4.** The onset potential of Ir dissolution determined from the dissolution rate vs. time diagrams presented in Figure 4 and S10.

| $c_{ethanol}$ / mM | pH | $E_{onset,anodic}$ / $V_{RHE}$ |
|---|---|---|
| 0 | 13 | 0.95 |
| 10 | | 0.95 |
| 100 | | 0.95 |
| 0 | 10.5 | 1.23 |
| 100 | | 0.72 |
| 0 | 8.5 | 1.25 |
| 100 | | 0.54 |
| 0 | 6.8 | 1.22 |
| 100 | | 0.53 |

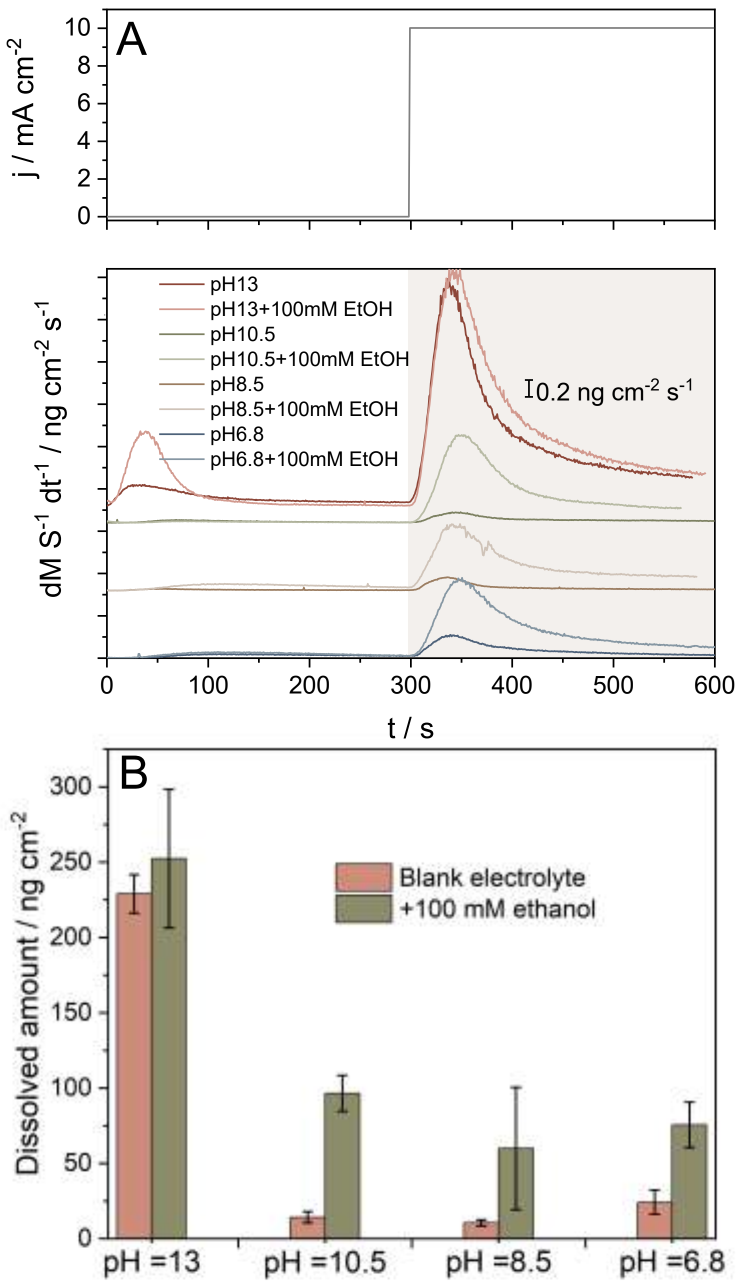


**Figure S11.** (A) Dissolution rates of Ir recorded with on-line ICP-MS while performing a galvanostatic electrochemical protocol. Two galvanostatic holds were performed, each for 300 s, the first one under open circuit conditions (j=0 mA cm$^{-2}$), the second one by applying 10 mA cm$^{-2}$ current density. (B) Dissolved amount of Ir calculated by integrating the dissolution rates presented in (A) highlighted with a light brown background. Error bars were calculated from at least two measurements, each performed on a pristine sample.

## References


(1) Karade, S. S.; Sharma, R.; Hedegaard, M. A. B.; Andersen, S. M. Stepwise Understanding on Hydrolysis Formation of the $IrO_x$ Nanoparticles as Highly Active Electrocatalyst for Oxygen Evolution Reaction. *Electrocatalysis* **2024**, *15* (4), 291–300. https://doi.org/10.1007/s12678-024-00874-x.

(2) Khalil, M.; Liu, N.; Lee, R. Super-Nernstian Potentiometric PH Sensor Based on the Electrodeposition of Iridium Oxide Nanoparticles. *Int. J. Technol.* **2018**, *9* (3), 446. https://doi.org/10.14716/ijtech.v9i3.1263.

(3) Mirbagheri, N.; Campos, R.; Ferapontova, E. E. Electrocatalytic Oxidation of Water by $OH^-$ - and $H_2O$-Capped $IrO_x$ Nanoparticles Electrophoretically Deposited on Graphite and Basal Plane HOPG: Effect of the Substrate Electrode. *ChemElectroChem* **2021**, *8* (9), 1632–1641. https://doi.org/10.1002/celc.202100317.

(4) Schalenbach, M.; Kasian, O.; Ledendecker, M.; Speck, F. D.; Mingers, A. M.; Mayrhofer, K. J. J.; Cherevko, S. The Electrochemical Dissolution of Noble Metals in Alkaline Media. *Electrocatalysis* **2018**, *9* (2), 153–161. https://doi.org/10.1007/s12678-017-0438-y.

(5) Zlatar, M.; Escalera-López, D.; Simon, C.; Briega-Martos, V.; Stojanovski, K.; Cherevko, S. PH Dependence of Noble Metals Dissolution: Iridium. *Electrochim. Acta* **2025**, *513*, 145450. https://doi.org/10.1016/j.electacta.2024.145450.

(6) Kasian, O.; Grote, J. P.; Geiger, S.; Cherevko, S.; Mayrhofer, K. J. J. The Common Intermediates of Oxygen Evolution and Dissolution Reactions during Water Electrolysis on Iridium. *Angew. Chemie - Int. Ed.* **2018**, *57* (9), 2488–2491. https://doi.org/10.1002/anie.201709652.

(7) Kasian, O.; Geiger, S.; Li, T.; Grote, J.-P.; Schweinar, K.; Zhang, S.; Scheu, C.; Raabe, D.; Cherevko, S.; Gault, B.; Mayrhofer, K. J. J. Degradation of Iridium Oxides via Oxygen Evolution from the Lattice: Correlating Atomic Scale Structure with Reaction Mechanisms. *Energy Environ. Sci.* **2019**, *12* (12), 3548–3555. https://doi.org/10.1039/C9EE01872G.

(8) Geiger, S.; Kasian, O.; Shrestha, B. R.; Mingers, A. M.; Mayrhofer, K. J. J.; Cherevko, S. Activity and Stability of Electrochemically and Thermally Treated Iridium for the Oxygen Evolution Reaction. *J. Electrochem. Soc.* **2016**, *163* (11), F3132–F3138. https://doi.org/10.1149/2.0181611jes.

(9) Cherevko, S. Electrochemical Dissolution of Noble Metals Native Oxides. *J. Electroanal. Chem.* **2017**, *787*, 11–13. https://doi.org/10.1016/j.jelechem.2017.01.029.

(10) Speck, F. D.; Zagalskaya, A.; Alexandrov, V.; Cherevko, S. Periodicity in the Electrochemical Dissolution of Transition Metals. *Angew. Chemie - Int. Ed.* **2021**, *60* (24), 13343–13349. https://doi.org/10.1002/anie.202100337.

(11) Marcel Pourbaix. *Atlas of Electrochemical Equilibria in Aqueous Solutions*, 2.;

National Association of Corrosion Engineers: Houston, Texas, USA, 1974.

(12) Cherevko, S. *Electrochemical Dissolution of Noble Metals*; Elsevier, 2018. https://doi.org/10.1016/B978-0-12-409547-2.13569-3.
